# Managing cognitive load in simulations: exploring the role of simulation technologists

**Published:** 2019-11-28

**Authors:** Matt Sibbald, Kyla Caners, Bingxian Wang

**Affiliations:** 1 Centre for Simulation-Based Learning, McMaster University, Ontario, Canada

## Abstract

**Background:**

Facilitating simulation is a complex task with high cognitive load. Often simulation technologists are recruited to help run scenarios and lower some of the extraneous load. We used cognitive load theory to explore the impact of technologists on instructors, identifying sources of instructor cognitive load with and without technologists present.

**Methods:**

Data were collected from 56 simulation sessions for postgraduate emergency medicine residents. Instructors delivered 14 of the sessions without a technologist. After each session, the instructor and simulation technologist (if present) provided quantitative and qualitative data on the cognitive load of the simulation.

**Results:**

Instructors rated their cognitive load similarly, regardless of whether simulation technologists were present. However, the composition of their cognitive load differed. Instructors experienced reduced cognitive load related to the simulator and technical resources when technologists were present. Qualitative feedback from instructors suggested real consequences to these differences in cognitive load in (1) perceived complexities in running the scenario, and (2) observations of learners.

**Conclusion:**

We provide evidence that simulation technologists can remove some of the extraneous load related to the simulator and technical resources for the instructor, allowing the instructor to focus more on observing the learner(s) and tailoring the scenario to their actions.

## Abstract (fr)

**Contexte:**

Faciliter la simulation est une tâche complexe qui comporte une charge cognitive élevée. Des technologues en simulation sont souvent recrutés pour aider à exécuter des scénarios et à alléger la charge extrinsèque. Nous avons utilisé la théorie de la charge cognitive pour explorer l’impact des technologues sur les instructeurs, en identifiant les sources de la charge cognitive de l’instructeur avec et sans la présence du technologue.

**Méthodes:**

Les données ont été recueillies à partir de 56 ateliers de simulation auprès des résidents en médecine d’urgence. Les instructeurs ont animé 14 de ces ateliers sans technologue. Après chaque session, l’instructeur et le technologue en simulation (s’il était présent) ont fourni des données quantitatives et qualitatives sur la charge cognitive associée à la simulation.

**Résultats:**

Les instructeurs ont évalué leur charge cognitive de façon similaire indépendamment de la présence du technologue en simulation. Cependant, la composition de leur charge cognitive était différente. Les instructeurs ont subi une moindre charge cognitive liée au simulateur et aux ressources techniques en présence des technologues. La rétroaction qualitative des instructeurs a suggéré des conséquences réelles liées aux différences de charges cognitives concernant (1) les complexités perçues en exécutant le scénario, et (2) les observations des apprenants.

**Conclusion:**

Nous fournissons des données probantes suggérant que les technologues en simulation puissent éliminer une partie de la charge extrinsèque liée au simulateur et aux ressources techniques, ce qui permet à l’instructeur de se concentrer davantage sur l’observation de l’apprenant et d’adapter le scénario à leurs actions.

## Introduction

Facilitating simulation is a complex task. Instructors often consider simulation sessions in three components: (1) pre-brief and briefing where learners are oriented, objectives are discussed, and a safe climate is established (2) conducting the actual scenario often requiring the manipulation of mannequins and confederates, and (3) debriefing where learner reflection is often facilitated by instructor observation, commentary and video replay.^1^ Running the scenario can be a particularly demanding task as instructors often have to divide their time between several different tasks (1) directing the flow of the scenario, (2) providing input to the mannequin and confederates, (3) observing the performance of the learner, (4) keeping track of time and objectives. In addition, preparing for various simulation sessions requires a large amount of set-up, and takedown. The turnover associated with this can create additional stress on the instructor. This demand on instructors has led to increasing use of simulation technologists during scenarios to assist in some of these tasks. However, the impact of simulation technologists during scenarios on the educational value of the simulation has not been well studied.

Cognitive load theory is a unique lens to view the demands on instructors when running a scenario.^2^^,^^3^ The fundamental assumption is that a finite amount of working memory is available to be divided into task-specific cognitive effort (intrinsic load), task- irrelevant cognitive effort (extraneous load) and residual working memory capacity, which potentially can be devoted to reflection-in-action for learning (germane load).^4^^,^^5^ While cognitive load was conceived as means of explaining the impact of instructional design decisions on learning, its principles are equally applicable to other performance based cognitive tasks, such as facilitating simulation. The primary goal, and therefore the intrinsic load of running a scenario, is to facilitate the learner meeting the learning objectives. This usually requires careful observation and attention of the learner, making modifications to the simulation to respond to their actions or inactions, redirecting it to the learning objectives as well keeping track of discussion points for debriefing. The technologic interface needed to manipulate the mannequin, troubleshoot difficulties or coordinate with the confederates could be viewed as an extraneous load to this primary goal. Familiarity with the equipment, confederates, scenario and environment can help modulate the degree of extraneous load these components of the simulation impose on the instructor.

Managing some component of this extraneous load through support of the simulation by technologists is appealing for several reasons. First, performance on monitoring tasks, like learner observation, are effortful and decay quickly over time, or with competing tasks. Removing some competing tasks from instructors may free more working memory room to devote to learner observation. Second, responding to learner action or inaction often requires a direct response by the mannequin or confederate (e.g., worsening oxygen saturations when supplemental oxygen is not provided) but also judgment about whether the scenario needs to be redirected (e.g., by having a passerby suggest intubation as a next step) so the learner can achieve the intended learning objectives. This judgment requires reflection-in-action by the instructor, a cognitively taxing process. Again, use of simulation technologists to free up instructor cognitive load may result in better learner achievement of learning objectives. Finally, learner feedback is often facilitated using formative assessment scales during scenarios (e.g. the Mayo teamwork scale).^6^ These scales frequently rely on tallying observable behaviors to help learners focus on their performance of particular non-technical skills. The significant cognitive load involved in keeping track of multiple observable behaviors, especially in multiple domains, has been documented.^7^^,^^8^ Instructors with fewer cognitive demands would have more cognitive load to devote to formative assessment.

While the addition of a simulation technologist can assist in all of the above functions, it adds an additional extraneous cognitive load on the instructor in coordinating a response. The instructor must now communicate with the simulation technologist, and coordinate responsibility for tasks during the scenario. This added cognitive load might be greater when the simulation technologist is not from healthcare background. According to a study published in 2015, close to 50% of simulation technologists working at various simulation centres are from non-healthcare backgrounds.^9^ The added cognitive load must be balanced against the reduction in cognitive load afforded by having the simulation technologist run the equipment.

The goal of this study was to describe the cognitive load of instructors and its sources and to quantify the effect simulation technologists co-facilitating a session have on the cognitive load of instructors. Importantly, simulation technologists add to the human resource cost of running simulations. In financially constrained environments without technologists, many instructors report challenges in simultaneously running a scenario and observing subtleties to support debriefing of learners. Therefore, determining whether technologists affect instructor cognitive load and observation capacity has important practical implications.

Research question 1: What are the sources of cognitive load among instructors and technologists running high fidelity simulation?

Research question 2: What is the impact of simulation technologists co-facilitating sessions on the cognitive load of instructors?

## Methods

### Study setting

The study took place within a longitudinal high- fidelity simulation curriculum in emergency medicine for postgraduate residents. Each year, there are twenty-four sessions, half for second year residents and half for fourth year residents. Learners participate in two cases each session. Four or five learners attend each session.

Scenario content for the second year residents focused on developing skills as a team leader and working through ambiguous patient presentations. Cases included a wide variety of content areas, such as trauma, all types of shock, pediatrics emergencies, and obstetrical emergencies. Content for fourth year residents focused on management skills of complex presentations, rare diseases, and difficult encounters. For example, one session is a two-patient trauma scenario. One of the patients requires a surgical airway while the other patient is loud and agitated due to hypoglycemia. Scenario topics are outlined in Table 1

**Table 1:** Scenario topics Location

Location	Learner Level	Topic	Technologist present
Simulation center	4^th^ year	Multi-Patient Trauma	YES
Hospital	2^nd^ year	Vital signs absent	NO
Hospital	2^nd^ year	Abdominal aortic aneurysm Adrenal crisis	NO
Simulation center	4^th^ year	Multi-Patient Trauma Obstetrical Trauma	YES
Simulation center	4^th^ year	Obstetrical resuscitation Neonatal resuscitation	YES
Hospital	2^nd^ year	Trauma	NO
Simulation center	2^nd^ year	Trauma	YES
Simulation center	4^th^ year	Pediatric scenarios	YES
Hospital	2^nd^ year	Altered level of consciousness Toxic alcohol ingestion	NO
Hospital	4^th^ year	Neurologic emergency Endocrine emergency	NO
Hospital	2^nd^ year	Ectopic pregnancy Burn victim	NO
Simulation center	2^nd^ year	Pediatric crisis Neonatal resuscitation	YES
Hospital	4^th^ year	Toxicology scenarios	NO
Simulation center	2^nd^ year	Laryngospasm Massive pulmonary embolism	YES
Hospital	4^th^ year	Respirology scenarios	NO
Simulation center	4^th^ year	Multi-Patient Trauma	YES
Simulation center	2^nd^ year	Vital signs absent	YES
Simulation center	4^th^ year	Multi-Patient Trauma Obstetrical Trauma	YES
Simulation center	2^nd^ year	Trauma	YES
Simulation center	4^th^ year	Pediatric crisis	YES
Simulation center	2^nd^ year	Pediatric crisis Neonatal resuscitation	YES
Simulation center	4^th^ year	Obstetrical crisis Neonatal resuscitation	YES
Simulation center	2^nd^ year	Ectopic pregnancy Burn victim	YES
Simulation center	4^th^ year	Cardiology scenarios	YES
Simulation center	2^nd^ year	Laryngospasm Massive pulmonary embolism	YES

Before this study, instructors did not have access to simulation technologists for these sessions. Our Centre provided simulation technologist support on a trial basis for complex scenarios in order to study their impact on instructors. When technologists were not present, instructors were required to setup and takedown all equipment. We surveyed instructors and simulation technologists about their cognitive load and sources of cognitive load when conducting scenarios.

All instructors and simulation technologists were familiar with and had used the high fidelity equipment for more than two years. The three simulation technologists involved all came from a healthcare background with more than four years of experience in healthcare simulation. The sixteen simulation instructors who participated in the study all had facilitated simulation previously.

### Recruitment and consent

We recruited all instructors and simulation technologists via email. All learners were asked a question on their routine anonymous feedback form about whether the data could be used for study purposes.

### Data sources

After each session, the instructor and simulation technologist (if present) completed a survey on the cognitive load of the simulation. Both quantitative and qualitative data were collected on the sources of cognitive load. Several faculty facilitators and all technologists were surveyed more than once.

Quantitative data included measurements of the overall cognitive load of running the scenario and the cognitive load attributed to different components (similar to the approach of Leppink^10^) using subjective rating scales.^11^^,^^12^ Components of the simulation contributing to cognitive load were identified through surveying three simulation technologists and two simulation instructors, followed by a focus group with the respondents to clarify and refine the categories. The sources of cognitive load identified through this process included: the learner, simulator(s), technical resources, confederate(s), fellow instructor(s) or technologist, scenario material. The questions took the format of “When running the scenario, how much mental effort did you have to devote to each?” with a sliding bar from “Did not think about it at all (1)” through to “Had to think so hard my brain hurt (7)”.

Qualitative data included answers to these five questions:

- What made the scenario complex?
- Did you encounter any specific challenges or concerns with the scenario (e.g., fire alarm going off, view of learner blocked, concerns about the equipment getting damaged, etc.)?
- Did you redirect or modify the scenario on the fly? If so, how?
- What was the most important observation you made of the learner?
- What would you change about the scenario for next time?

We based the questions on the thematic analysis of a focus group involving two simulation instructors and three simulation technologists, whom we asked about cognitive load running scenarios and its potential impact.

### Analysis

For the first research question, descriptive statistics (mean and standard deviation) were used to describe cognitive load among instructors and technologists for the sessions where both were present. Cognitive load of instructors was compared with that of technologists using Mann-Whitney U tests for non- parametric ordinal data^13^ (SPSS version 21, IBM). In order to maintain an overall type I error rate of 0.05, Bonferroni correction for multiple comparisons was applied.^14^ For the second research question, the cognitive load of instructors was compared when technologists were and were not present using Mann- Whitney U tests for non-parametric ordinal data with Bonferroni correction for multiple comparisons.

We planned a sample size of 36 sessions, which gave an 80% power to detect a 20% difference between groups (http://powerandsamplesize.com) as previous literature has identified performance variation associated with cognitive load differences of 20- 25%^15^^,^^16^.

Three independent researchers (MS, KC and BW) each analyzed the qualitative survey responses for each of the five questions using thematic analysis by. Each researcher independently reviewed the responses and identified 2-3 key themes via inductive coding using a realist paradigm, comparing across different groups.^17^^,^^18^ Themes were iteratively reviewed and distilled into the written report, allowing consensus to emerge.

The Hamilton Integrated Research Ethics Board provided ethics approval.

## Results

Data were collected from 56 simulation sessions each facilitated by one of the 16 different instructors. The instructors delivered the simulation sessions without a technologist present in 14 of the 56 sessions. Simulation technologists provided feedback for 20 of the 42 sessions when they assisted.

### Instructor cognitive load compared to simulation technologists

The overall rated cognitive load of instructors and simulation technologists was similar (Table 2), however, instructors perceived the sources of cognitive load differently than technologists. Instructors perceived less cognitive load related to the simulator, technical resources, confederate, and scenario material (all mean differences greater than - 1.11, p<0.05). The instructors perceived similar cognitive load related to learners and scenario complexity as technologists (p not significant).

**Table 2:** Cognitive load of instructors and technologists

	Instructor without technologist present	Instructor with technologist present	Technologist	P value comparing instructors with and without a technologist	P value comparing instructors to technologists
How much mental effort did you need to devote to running the scenario?	4.43 ± 1.70	3.69 ± 1.52	4.40 ± 1.34	0.56	0.72
How much mental effort did you need to devote to each of the following:					
The learner	4.69 ± 0.48	4.85 ± 0.84	4.78 ± 1.17	0.24	1.0
The simulator(s)	4.73 ± 1.01	3.00 ± 1.50	4.56 ± 1.55	**0.008**	**0.02**
The technical resources	4.70 ± 1.16	2.88 ± 1.49	4.20 ± 1.27	**0.008**	**0.02**
The confederate(s)	3.63 ± 0.74	3.39 ± 1.39	4.50 ± 1.36	1.0	**0.05**
Fellow instructor(s) or technologist	3.20 ± 1.03	3.34 ± 1.38	4.29 ± 1.53	1.0	0.24
The scenario material	2.75 ± 1.06	3.31 ± 1.58	4.72 ± 1.07	1.0	**0.03**
Rate the scenario complexity	3.57 ± 1.34	4.50 ± 1.38	4.55 ± 1.23	0.30	1.0

### Instructor cognitive load with and without simulation technologists

Instructors rated their cognitive load similarly regardless of whether simulation technologists were present (Table 1). However, the composition of their cognitive load differed. Instructors experienced less cognitive load related to the simulator and technical resources (mean differences -1.73± 0.48 and -1.82± 0.51 respectively, both p<0.01) when simulation technologists were present.

### Thematic analysis

Salient thematic differences emerged in instructor responses to the 5 post simulation questions, based on whether or not a technologist was present, described below.

**What made the scenario complex?** When technologists were not present, instructors commented on the complexities of the medical content of the scenario, particularly around recognizing severe illness states or important management steps.

*“The team had to recognize the toxidrome (which required some prompting from the confederate due to mannequin limitations) and then recognize the associated dysrhythmias. This meant changing vitals frequently on the mannequin in addition to speaking on behalf of an awake patient while also trying to ensure the confederate nurse was following appropriate cues.”* —Instructor

When technologists were present, both instructors and technologists commented on the challenges of observing and responding to learners (especially when multiple learners were present), communicating with each other and the confederates, and controlling the flow of the scenario when unanticipated action or inaction occurred.

*“It's almost impossible to be able to listen to all things you need to at the same time. Let alone respond appropriately without missing something along the way”* —Technologist

*“There were a lot of bodies in the room, so it was quite challenging to hear and to coordinate all the pieces... I essentially served as the ‘coordinator’ while the other two instructors each observed one of the patients.”* —Instructor

**Did you encounter specific challenges?** When instructors managed simulations without technologists, they described technical issues in more than two thirds of cases including programming malfunctions, mannequin malfunctions, and managing unanticipated leaner actions that required technical intervention during the scenario.

*“Despite having pre-programmed the case for the session, the SimPad was running a different case. I had to stop part way through and try re-loading to make sure that I had selected the right case. It seems it was a glitch with the SimPad. It kept running the wrong case, which meant lots of on the fly adjustment of vital signs. This made it much harder to observe the learner actions.”* —Instructor

In contrast, when technologists were present, challenges cited by both technologists and instructors related to obstructed view of the learners, noise level in the room, and coordinating with confederates.

*“View of learner blocked, difficult to hear learner voices as multiple learners speaking at the same time, noise from the compressor, IV pump alarm, and multiple faculty speaking in control room”* – Technologist

**Did you redirect the scenario on the fly?** Without technologists, instructors described five instances of redirecting for technical reasons (e.g. mannequin lost a pulse but was not supposed to) and one redirection because of learner actions. When technologists were present, instructors describe redirecting scenarios only for unanticipated learner actions or inaction. Technologists described one behind-the-scenes adaptation for a mannequin not working and multiple modifications to help learners realize an incorrect action.

*“Decreased sats (oxygen saturations) on fly, as the sensor on mannequin indicated bagging rate was ineffective, feedback provided to resident via confederate about rate, resident increased bagging rate and sats resolved.”* —Technologist

**What was the most important observation you made of the learner?** Observation comments were exclusively related to global impressions (largely around team coordination, organization and leadership) when technologists were not present, with only one specific observation moment noted.

*“The team was extremely calm and coordinated. The team leader, in particular, was extremely clear in the management of the case and shared her logic clearly with the team.”* —Instructor

In contrast, when technologists were present, over half of the observations related to specific medical content or observations.

*“The team leader developed fixation error around the hypotensive trauma patient. Without help from his team, he was unable to identify other possible causes of hypotension.”* —Instructor

Technologists commented on learner ability to ‘suspend disbelief’, notice simulated cues, and the perception of a learner being overwhelmed.

*“They did NOT suspend reality. There were a couple times where they did not complete a task because they weren't sure they could perform it on the mannequin without causing harm.”* —Technologist

**What would you change about the scenario for next time?** When technologists were not present, instructors considered ‘dry-runs’ and reprogramming.

*“I would make sure the programming is running well!”* —Instructor

When technologists were present, instructors commented on improving realism, advanced planning to manage issues with confederates, and adapting the scenario to meet the learning objectives more effectively.

*“I would ensure that the confederate nurse was better prepared to emphasize the CHF and crackles. I would also make sure that the patient's voice was portrayed as awake but confused rather than as grunting and barely responding. It also may be worth changing the case to make it clearer the patient is in thyroid storm. This presentation was a rare one - perhaps a more “common” version of this already rare presentation would lead to better accomplishing the scenario objectives.”* —Instructor

Technologists discussed changing the scenarios to reduce complexity as well as optimizing sound quality.

## Discussion

Cognitive load theory provides a unique perspective in understanding the challenge of running high fidelity simulations. Both instructors and simulation technologists have multiple competing demands on their attention while running a scenario. This study provides insight with both quantitative and qualitative data on these varied demands. Instructors and technologists perceived similar cognitive load related to running a simulation. Sources of this cognitive load included the learner, simulator, technical aspects of the simulation, confederate, fellow instructor or technologist and the scenario material itself.

Simulation technologists affected the types of cognitive demands instructors faced. When technologists were present, the instructor’s cognitive load related to the simulator and technical resources were reduced. This is consistent with our hypothesis that simulation technologists can manage some of the extraneous cognitive load related to the equipment. Qualitative feedback from instructors suggested real consequences to these differences in cognitive load in (1) perceived complexities in running the scenario, and (2) observations of learners. When technologists were not present, instructors frequently described specific technical challenges (equipment, programming and mannequin malfunctions), and focused observations on global team function. In contrast, when technologists were present, the instructors described challenges related to observing and coordinating rather than running the scenario; and described more often content-based, specific observations of learners. Whether or not these differences translate into enhanced learner value or behavioral change remains to be established. Nevertheless, the data presented in this study add to the argument that the presence of technologists favorably affected the quality of instructor observation. This finding may help justify the additional cost of technologists.

Interestingly, we uncovered differences between technologists and instructors in sources of cognitive load. Technologists perceived greater cognitive load related to confederates, fellow instructors and scenario material than instructors. This increased load was present even when compared to instructors running scenarios solo without technologists. We do not think this relates to familiarity with the simulation environment or experience-running simulations, as all the technologists in this study were likely to have run far more scenarios than the instructors did. However, instructors may have a more intuitive feel of the scenario content domain, and different relationships with confederates and fellow instructors by virtue of their greater experience in clinical contexts. In contrast, technologists are not necessarily content experts, therefore may need to devote more mental effort to the scenario material. Alternatively, instructors may place less emphasis on these components of the simulation. The impact of these findings is unclear, and may benefit from further study.

This study has several strengths including its mixed method approach, and sampling of different levels of learners. However, there are several important limitations to consider in interpreting our findings. First, the allocation of technologists was not randomized. Technologists were more frequently used in more complex scenarios, such as concomitant management of multiple patients, multiple confederates and multiple learners. This would tend to understate the differences between instructor cognitive load with and without technologists. Replication of these findings in a randomized study will be important to verifying their magnitude and importance. Second, our instructors were very experienced and many were involved in writing the scenarios. This experience and familiarity might mitigate some of the cognitive load experienced in running a simulation without a technologist, further reducing the differences we identified. Third, the sample involved a limited number of instructors and technologists. Fourth, while the scenario content varied widely, it all related to postgraduate emergency medicine training. While it is unlikely that the differences in cognitive load found in this study are context specific, replication in other clinical settings would be important. Finally, the measure of cognitive load involved a standard Paas scale, but used novel anchors “Did not think about it at all (1)” and “Had to think so hard my brain hurt (7)” which had not been formally validated.

### Conclusion

Cognitive load theory provides insight into the complexities of running simulation. We provide evidence that simulation technologists can remove some of the extraneous load related to the simulator and technical resources for the instructor, allowing the instructor to focus more on observing the learner(s) and tailoring the scenario to their actions.
